# The impact of COVID-19 on the changes in health behaviours among Black, Asian and Minority Ethnic (BAME) communities in the United Kingdom (UK): a scoping review

**DOI:** 10.1186/s12889-023-15978-0

**Published:** 2023-07-31

**Authors:** Ifunanya Nduka, Isabella Kabasinguzi, Nasreen Ali, Peter Ochepo, David Abdy, Erica Jane Cook, Chimeme Egbutah, Sally Cartwright, Gurch Randhawa

**Affiliations:** 1 Institute for Health Research, University of Bedfordshire, Luton, UK; 2 School of Psychology, University of Bedfordshire, Luton, UK; 3 Luton Borough Council, Luton, UK

**Keywords:** Covid-19, BAME, Health behaviors, Change, UK

## Abstract

**Background:**

The COVID-19 pandemic has led to changes in health behaviours, which include eating patterns and nutrition, smoking, alcohol consumption, sleeping patterns, physical activity and sedentary behaviour. There is a dearth of evidence reporting the impact of COVID-19 on the health behaviour of Black, Asian and minority ethnic (BAME) communities. This scoping review synthesises the available evidence on the impact of COVID-19 on the changes in health behaviours among BAME communities in the UK.

**Methods:**

Following a keyword strategy, 16 electronic databases were searched for articles that met the screening criteria. These articles were then reviewed in full text. Empirical studies that assessed COVID-19 related health behaviour changes among BAME communities in the UK, conducted during the COVID-19 pandemic between July 2020 and August 2021 and published in English language, were set as inclusion criteria. An initial 2160 studies were identified in the selected databases. After removing duplications and screening the title and abstracts of the 2154 studies, only 4 studies were selected to be reviewed as they met the inclusion criteria. The included studies employed different sample sizes which ranged from *N* = 47 to *N* = 30,375 and reported several health behaviour changes. Out of the 4 included studies, 3 studies included BAME groups within their sample as a subgroup while one study focused specifically on BAME groups.

**Results:**

The scoping review found that there were lower levels of physical activity among BAME groups compared to the White ethnic groups. About 41.7% of BAME groups reported drinking less alcohol than usual compared to their white counterparts who were, 34% of whom reported drinking less alcohol. Study participants from BAME backgrounds had the greatest effect of COVID-19 on decisions to purchase healthier food compared to people from white backgrounds whose decisions on purchasing healthier food were least affected. Similar to other ethnic groups the COVID-19 pandemic increased positive hygiene among BAME groups which is important in preventing other diseases and infections. .

**Conclusion:**

COVID-19 had a significant impact on the health behaviours of BAME groups especially during the lockdowns as they reported changes to behaviour such as low levels of physical activities. Hence, it is important to promote health awareness among BAME groups to encourage healthy living. In addition, programmes such as physical fitness activities that favour BAME groups should be put in place, for example BAME women’s walking groups to encourage people from BAME backgrounds to engage more in physical activities. Furthermore, healthy food programmes such as food parcels can be given to people from BAME backgrounds who are not able to afford healthy food due to the impact of COVID-19. Similar to other ethnic groups, the COVID-19 pandemic has increased positive hygiene among BAME groups which is important in preventing other diseases and infections.

**Supplementary Information:**

The online version contains supplementary material available at 10.1186/s12889-023-15978-0.

## Introduction

The COVID-19 pandemic has led to changes in health behaviours and consequently, health outcomes [1]. Health behaviours are intentional or unintentional actions or habits taken by individuals that affect health or mortality [2]. Health behaviours include physical activity, smoking, alcohol use, sleep, diet, healthcare-seeking behaviours, adherence to medical treatments and sedentary behaviours [2, 3]. Changes in health behaviour through disruptions to daily routines such as reduced physical activity and COVID-19 related anxiety impact on health and wellbeing [3, 4].

Before COVID-19, a decrease in preventative health behaviours accounted for 40% of the causes of premature mortality [4]. According to the World Health Organisation (WHO), health behaviours and lifestyle factors are a major contributory factor towards non-communicable diseases such as diabetes and cardiovascular diseases [5, 6]. In addition, epidemiological studies have reported that preventative health behaviours can delay the risk of premature mortality and morbidity [7]. Furthermore, the combined effects of four defined health behaviours (smoking, alcohol consumption, fruit and vegetable intake and physical activity) predicts a four-fold difference in the risk of dying over an eleven year time period for older and middle-aged people [8].

Available evidence argues that the COVID-19 social distancing measures including the two lockdowns on March 2020 and November 2020 [9], have affected health behaviours [10, 11] with social distancing having a substantial impact on decreased physical activity [12–14], dietary choices, specifically the consumption of less healthy foods such as high sugar calorie-rich foods [15–17], increase in alcohol and tobacco consumption [18–21] and decrease in overall emotional wellbeing [22]. Sedentary behaviours such as prolonged sitting and screen time, has also increased due to social measures and constraints resulting from COVID-19 [1]. Studies have suggested that prolonged sedentary behaviours may increase hormonal dysfunction and cardiometabolic risks, which may aggravate underlying chronic conditions [23, 24]. Physical inactivity, as well as sedentary behaviours are independent determinants for poor cardiometabolic health [25]. Sedentary behaviours such as sitting time increases the susceptibility of cardiovascular diseases and mortality [3, 10].

Negative changes in health behaviours are likely to interact in complex ways. For example, those smoking tobacco may also be likely to drink more alcohol and engage in less physical activity [26]. These interacting behaviours are very likely to be associated with existing multiple disadvantage and health inequalities, such as lower socioeconomic status [27, 28]. The literature that discusses BAME communities and health behaviours focusses on the differences in preventive health behaviours between people from BAME backgrounds and people from white ethnic backgrounds during COVID-19 [29]. For instance, a study exploring changes during the COVID-19 lockdown reported that a higher percentage of people from BAME backgrounds than those from a white ethnic background said they would not take the COVID-19 vaccine [30].

BAME groups have been disproportionately affected by COVID-19 [29]. We are aware that the term BAME is contested and criticised because it risks homogenising the experience of people from different ethnic backgrounds and masking inequalities between groups; it also maintains white British ethnic identity as the dominant privileged norm [31]. We recognise that BAME groups are not homogeneous, and that individuals from these communities have their own narratives [32]. The disproportionate impact on BAME groups have been attributed to an intersection of environmental, physiological, deprivational as well as health-related behaviours [33].

Comparing ethnic differences in health behaviour during COVID-19, the literature highlights that BAME groups have shown irregular sleep patterns, decreased physical activity and less consumption of fruits and vegetables [34] than people from white ethnic groups [35]. Sales figures for alcohol indicate an overall increase in the consumption of alcohol [36–38]. BAME groups show lower levels of alcohol consumption during COVID-19, compared to their white counterparts [35]. An explanation for the low alcohol intake noted among people from BAME group during COVID-19 is that this may be due to social norms among BAME communities [39]. Evidence suggests that the differences in health behaviours may be associated with the disproportionate impact of COVID-19 on BAME groups [29]. This has caused increasing concerns on the long-term effects of negative health behaviour changes on mental and physical health outcomes [40].

There is limited evidence on the impact of COVID-19 on health behaviours among BAME groups [41, 42]. Therefore, this scoping review aimed to synthesise the evidence on the impact of COVID-19 on the changes in health behaviours and the lifestyle of BAME groups in the UK. The findings from this scoping review are important for policy makers, local councils, and commissioners to develop and commission programmes and interventions that will support the BAME communities improve health and wellbeing and tackle health inequalities.

## Methods

We conducted a systematic scoping review of published peer-reviewed articles reporting the impact of COVID-19 on health behaviours among BAME communities. We followed the methodological framework outlined by Arksey and O’Malley [43] for scoping reviews to guide our response to the research question of “What are the COVID-19 related health behaviour changes among BAME communities in the UK?”. The framework included identifying relevant studies, selecting studies, charting/extracting the data, collating, summarising, and reporting the results. Scoping studies are used to provide in-depth or comprehensive coverage (breadth) of available literature. Following the literature search on the COVID-19-related health behaviour changes among BAME communities, it was clear that there is insufficient evidence assessing and synthesising health behaviour changes among BAME groups. As a result, this scoping review focused on the breadth of relevant literature. Further, due to inadequate evidence in the previous search for literature, it was expected that the final included studies for this review were going to be heterogeneous, thus, the need for a scoping review.

## Identifying the relevant studies

### Search strategy

The search strategy was developed according to the research question and definition of key concepts. Relevant keywords were created under the categories of population (BAME group), region, COVID-19 and outcome (health behaviour changes). Search terms which were alternative words to keywords were formulated. These are presented in supplemental file 1. The search terms were combined with Boolean operators to search for empirical studies published between July 2020 and August 2021, in electronic databases. The Librarian was consulted in formulating search strings. Relevant electronic databases were identified using the University of Bedfordshire digital library under healthcare-related databases. These databases included AMED, British Nursing database, CINAHL plus with full text, Medline, PsycINFO, Pubmed central, Cochrane library, TRIP database, UK Pubmed central, socINDEX, Annual reviews, ISI Web of Science, Academic search complete, Credo reference, Sage premier and Scopus. In addition, the search process was extended to other website platforms such as Google and Google scholar. Reference lists of included studies were also searched manually (snowballing) for more relevant papers until a saturation point was reached where no new papers were being identified.

### Selecting the studies

The inclusion criteria applied included papers that assessed COVID-19 related health behaviour changes, papers that assessed BAME communities, papers written in English language, papers conducted in the UK, papers conducted during COVID-19. The exclusion criteria applied were papers that were not written in English language, papers that were conducted outside the UK, and papers that were not conducted during COVID-19.

**Fig. 1:**
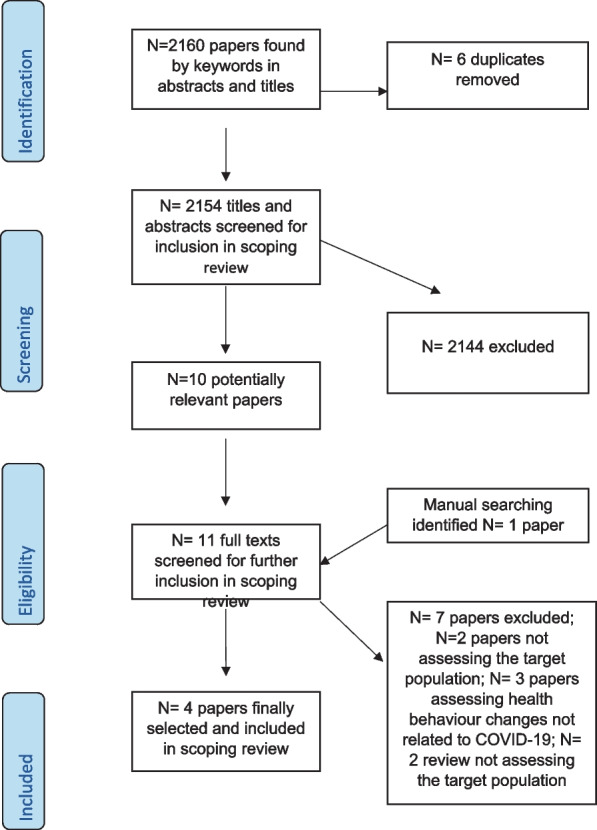
PRISMA flow diagram showing the study selection process

At first, the selected studies were screened by their title and abstract. This resulted in removal of papers that did not meet the inclusion criteria. Duplicates were also removed. Finally, the relevant papers were screened by their full texts and final decisions were made by the researchers on the final papers to be included in the review. A flow diagram showing the different stages of study selection based on PRISMA, is shown in Fig. 1.

### Data extraction

After the selection of the final studies, a data extraction spreadsheet based on the Cochrane data extraction was used at this stage of the scoping review. The data extraction spreadsheet was refined and developed to accommodate relevant information related to the research question of this scoping review. Thus, enabling the provision of a comprehensive and systematic extraction of information [44]. The data extraction spreadsheet included the following headings: date, authors, article number, publication type, description, methods, design, setting, outcomes (health behaviour changes) and bias identified. The data were extracted manually into the data extraction spreadsheet template after it was created and designed using Microsoft Word by IN. The data extraction process was reviewed by all authors while considering the scoping review research question and objectives. Disagreements were resolved through discussion and consensus and an agreement for final inclusion was reached by all authors.

### Charting and summarising the findings

A descriptive-analytical narrative method [43] was used to chart the findings of this scoping review. A spread sheet was developed using Microsoft excel to systematically chart the findings across the heterogeneous studies. This charting process involved extracting information from the included studies in line with the objectives of the scoping review. Therefore, the charting process recorded the health behaviour changes of BAME groups, the authors, year of publication, study setting and methods, participants and other findings.

## Results

Table 1 shows the features of the final included study articles. The study articles were all published in 2021, which could be attributed to the recent status COVID-19. The study articles included participants ranging from *N* = 47 to *N* = 30,375. Three study articles were quantitative studies [45–47] while one study article was a qualitative study [48]. All study articles recruited participants and collected data through online platforms such as Zoom, Microsoft Teams and application software, due to the COVID-19 restrictions. The employed research approaches were online cross-sectional surveys [45–47], secondary longitudinal and exploratory analysis [47] and in-depth qualitative study [48]. Furthermore, 3 study articles reported changes in individual health behaviours while one study article reported generally on changes in health behaviour among Muslim communities [48]. The individual health behaviour changes that were reported are as follows; physical activity [47], alcohol drinking behaviour [45] and eating and purchasing behaviours [46].

**Table 1:** The features of the final included study articles

S. no	Authors	Aim	Method	Participants (N)	Main findings
1	Wickersham *et al.* (2021) [47]	To describe the longitudinal trajectories of physical activity following the start of lockdown among students at a large UK university, and to investigate whether these trajectories varied according to age, gender and ethnicity	Secondary, longitudinal, and exploratory data analysis of the RMT King’s Move physical activity tracker app	770 (129 = White, 7 = Black, 77 = Asian, 21 = mixed, 31 = others)	-Lower levels of physical activity in BAME groups -Gradual increase in the number of steps walked per week following the commencement of the UK nationwide lockdown -Decrease in the number of miles run by females with no change among males.
2	Garnett *et al.* (2021) [45]	To assess what factors were associated with reported changes to usual alcohol drinking behaviour during the start of lockdown in the UK	A cross-sectional analysis of baseline survey data from the University College London (UCL)	30,375	-41.7% of BAME groups reported drinking less alcohol than usual. 34.0% of White groups reported drinking less than usual -37.1% of BAME groups reported drinking more than usual, 35.1% white groups reported drinking more than usual -BAME groups were more likely than those of white ethnicity to be drinking less than usual -Significant association between drinking and being younger, female, high income, stress about catching or becoming ill from COVID-19 and having an anxiety disorder -Psychological factors predicted changes in drinking behaviours
3	Ogundijo, D. A., Tas, A. A. and Onarinde, B. A. (2021) [46]	To measure the impact of COVID-19 on the eating and purchasing behaviours of people living in England based on sociodemographic variables	An online survey using questionnaires	911 (77 = Asian/Asian British, 38 = Black/Caribbean, 20 = mixed, 638 = White, 8 = Arab, 11 = others)	-BAME groups had the greatest effect of COVID-19 on decisionmaking and purchasing of healthier foods compared to participants from white backgrounds - Among the BAME groups, a considerable number of people from mixed or multiple ethnic groups had the lowest number of participants who had their decisions on healthier food affected “moderately or a little bit”
4	Hassan, S. M. *et al.* (2021) [48]	To understand better, perceptions of risk and responses to COVID-19 of members of the Muslim community living in the Northwest of England, and to understand the facilitators and barriers to adherence to restrictions and guidance measures	An in-depth qualitative study using interviews and focus group discussions	47	-There were changes reported in the overall health behaviours of the participants -Positive hygiene practices and social distancing were reported among some participants -Participants also described additional precautions they were taking to reduce risk of transmission, including wearing face masks/covering (well before this became mandatory), wearing gloves, using hand sanitisers and disinfecting food packaging before putting it away

## Discussion

We synthesised the evidence on the impact of COVID-19 on the changes in health behaviours among BAME groups in the UK and we recorded the different methodologies participants and findings used in included studies. The included studies showed that people from BAME groups did make up part of the study sample in each study but only one study focused specifically on BAME groups [4] and thus there is a paucity of research addressing COVID-19 related health behaviour changes among the BAME communities in the UK.

The included studies showed both positive and negative changes in health behaviours and lifestyle patterns due to COVID-19 among BAME population.

Only one of the included studies [47] reported changes in physical activity and showed lower levels of physical activity in BAME population when compared to white population. This is not uncommon, as studies conducted in other countries have also reported negative changes in physical activity during the COVID-19 lockdowns. In a cross-sectional survey in Zimbabwe, it was reported that more than half of the participants reduced their physical activity during COVID-19 [49]. Similarly, Rodriguez-Perez *et al.* [50] highlighted that an estimated 60% of their participants reduced their levels of physical activity. This may be explained by evidence that describes an increase in screen time [49], working from home or closures and restrictions on gym centres and sporting activities during the COVID-19 lockdowns [51]. Furthermore, a survey on US students comparing data from 2018/2019 to data collected during the 2020 lockdown has shown decreased levels of physical activity showing the negative effect of COVID-19 lockdown on young people [52].

According to Garnett *et al.* [45], people from BAME groups were more likely to drink less alcohol than usual during the COVID-19 lockdowns. Some BAME groups may drink less because of religious prohibitions. Likewise, in a multi-national survey which included BAME sample, Ammar *et* *al.* [53] found that a reduction in binge drinking was the major dietary habit change during the COVID-19 lockdown. Although Ammar *et* *al.* [53] suggests that younger people drank during the COVID-19 lockdowns due to reduced social interaction, Garnett *et al.* [45] reports that drinking more was independently associated with being younger. Furthermore, this scoping review reports that psychological stress projected changes in drinking behaviours. This stress could be associated with fear of contracting COVID-19 and becoming severely ill or low finances due to the associated economic loss. While stress is a risk factor for the inception of alcohol misuse, it can also act as a polarising factor for people to reduce alcohol intake and improve health [54].

Changes in eating and purchasing of healthier foods among BAME group was reported by one of the studies we reviewed [46]. Although the reason for the changes is not clear, it may be due to cultural, social or economic factors [55]. Pietrobelli *et al.* [56] identified an increase in the consumption of fruits among 41 children in Italy. This is similar in four other studies [50, 57–59]. This can be attributed to an increase in home cooking due to lockdown restrictions as well as WHO guidelines on the consumption of fruits and vegetables during lockdown [40, 60]. However, other studies globally have shown decrease in the consumption of fresh foods [40, 61]. A survey in Zimbabwe attributed this decrease to increased price and unavailability of fresh foods due to lockdown restrictions [49]. Another study in India reported that 32% of their respondents had indicated that an increase in price was a reason for their reduced intake of fruits and vegetables [62]. Nonetheless, there are very limited empirical evidence-based studies measuring the effects of COVID-19 on the dietary behaviours among BAME group in the UK.

This scoping review has further revealed the increase in protective health behaviours such as positive hygiene practices and social distancing. A possible explanation for these behaviours could be a means to mitigate risk of contracting COVID-19. Several studies have established links between risk perceptions and protective health behaviours during pandemics. A study in Italy that explored the association between risk perceptions and compliance with recommendations during the 2009 Influenza H1N1 pandemic, reported that participants complied with recommended behaviours due to their perceived risk of contracting the virus [63]. A review of demographic and attitudinal determinants of protective behaviours during a pandemic further revealed that higher levels of perceived risk and severity of disease are associated with adoption of recommended behaviours in a pandemic [64]. Conversely, an American study has found out that, while perceived risk of contracting COVID-19 may increase the level of protective health behaviours, perceived severity of COVID-19 did not [65]. Furthermore, an international study has found out that perceived risk of COVID-19, perceived severity of COVID-19 and trust in government were of little importance in voluntary compliance of protective health behaviours.

Nevertheless, this scoping review has shown that there is scarce evidence on health behaviour changes among BAME groups during the COVID-19 pandemic. Smoking, high alcohol consumption, physical inactivity and a poor diet are four principal behavioural risk to health with the latter two also causing obesity [66]. Their prevalence varies across the population, although prevalence is highest in more deprived communities [33]. Evidence also shows that these behavioural risks account for two-thirds of the incidence of cardiovascular diseases, chronic conditions, diabetes and cancer [67–69]. The existing inequalities on ethnic minorities following the COVID-19 pandemic, is now well reported. Available evidence has suggested that the cause of these inequalities is an intersection of deprivation, environmental, cultural, behavioural and physiological factors [33]. COVID-19 has highlighted the health inequalities experienced by ethnic minorities, thus, there is an urgent need to prevent and manage ill health in ethnic minority communities.

## Strengths and limitations of the study

To the best of our knowledge, this is the first scoping review that aimed to explore the impact of COVID-19 on the changes in health behaviours among BAME groups in the UK. The search strategy for this scoping review was constrained to articles published in English language due to the UK context of the research question. Also, this scoping review did not appraise the quality of the evidence in the primary studies as is customary in systematic reviews. As a result, the validity and methodological quality of the included studies are not known. Further, only four studies met the inclusion criteria and were included for this scoping review. Consequently, findings from this scoping review are very limited and may not be generalised. Therefore, there is need for further research to explore the experiences and health behaviour changes among ethnic minorities in the UK following the recent COVID-19 pandemic.

## Conclusion

The COVID-19 pandemic had a significant impact on the health behaviour of BAME groups especially during the lockdowns. Research evidence has reported changes in eating habits and the purchasing of healthier foods, but low levels of physical activities. Hence, there is need to promote health awareness among BAME groups to encourage healthy living particularly the importance of maintaining an active lifestyle. In addition, programmes such as physical fitness activities that favour BAME groups can be put in place, for example BAME women’s walking groups, men’s walking groups and young people’s exercise groups to encourage people from BAME backgrounds to engage more in physical activities. Healthy food programmes, such as giving out healthy food vouchers and parcels can be distributed to people from BAME backgrounds who are not able to afford healthy food due to the impact of the COVID-19 pandemic. Similar to other ethnic groups the COVID-19 pandemic increased positive hygiene among BAME groups which is important in preventing other diseases and infections. This scoping review has highlighted that there is limited evidence on the impact of COVID-19 on health behaviour of BAME communities living in the UK. Hence, there is a need for further research to explore COVID-19 related health behaviour changes among BAME communities.

### Supplementary Information

**Additional file 1.** 

## Data Availability

All data generated or analysed during this study are included in this published article [and its supplementary information files].
