# Supplementary material for: The impact of COVID-19 on the changes in health behaviours among Black, Asian and Minority Ethnic (BAME) communities in the United Kingdom (UK): a scoping review
Source: BMC Public Health. 2023 Jul 31;23:1466. doi: 10.1186/s12889-023-15978-0 (PMC10391900; doi:10.1186/s12889-023-15978-0)
Supplement: Supplementary file 1 — Additional file 1. [file 12889_2023_15978_MOESM1_ESM.docx]

**Supplementary Information**

**The impact of COVID-19 on the changes in health behaviours among Black, Asian and Minority Ethnic (BAME) communities in the United Kingdom (UK): a scoping review”**

| **Additional file 1- Search terms** |
| --- |
| (Bame OR Bme OR Black OR ethnic OR Asian OR Pakistan* OR Bangladesh* OR “minority ethnic” OR minority OR Africa* OR Chinese) AND (Coronavirus OR Covid-19 OR 2019-ncov OR Sars-cov-2 OR Cov-19) AND (UK OR “United Kingdom” OR Britain OR England OR Scotland OR Wales OR “Northern Ireland”) AND (change OR modification) AND ((“physical activity” OR exercise OR running OR walking OR cycling OR swimming OR sports) OR lifestyle OR (smoking OR “tobacco consumption” OR “substance use”) OR (diet OR “food pattern” OR nutrition) OR “sleep pattern” OR “sedentary behaviour”) |
